# Green synthesis of a lactoferrin-infused silver nanoparticle gel for enhanced wound healing

**DOI:** 10.1038/s41598-025-94450-y

**Published:** 2025-04-30

**Authors:** Ahmed K. Geneidy, Maii A. Abdelnaby, Doaa A. Habib, Heba M. Elbedaiwy, Kamel R. Shoueir

**Affiliations:** 1 Department of Pharmaceutics, Faculty of Pharmacy, Damanhour University, Damanhour, Egypt; 2 Department of Pharmacy Technology, Faculty of Technological Health Sciences, Borg Al Arab Technological University, Alexandria, Egypt; 3 Ministry of Health and Population, Health Affairs Directorate, Alexandria, Egypt; 4 Department of Pharmaceutical Technology, Faculty of Pharmacy, Alsalam University, Kafr El Zayat, Egypt; 5 Institute of Nanoscience & Nanotechnology, Kafrelsheikh University, 33516 Kafrelsheikh, Egypt

**Keywords:** Lactoferrin, Carbopol gel, AgNPs-LTF, Antibacterial, Antioxidant, And wound healing, Drug delivery, Pharmaceutics

## Abstract

The study analyzed the benefits of nano-silver (AgNPs) in reducing side effects and enhancing efficacy, highlighting the advantages compared to silver ions. The study examined the production of AgNPs-lactoferrin complexes (AgNPs-LTF) using bovine lactoferrin (LTF) at 1, 2, and 4 mM concentrations. The objective was to create an AgNPs-LTF gel with Carbopol as the base and assess its effectiveness in enhancing wound healing in rats. UV–Vis, PL, FTIR, and XRD analyses confirmed the synthesis of AgNPs. Microscopic examinations (TEM and SEM) showed mainly spherical AgNPs in the AgNPs-LTF samples, with diameters between 11 and 27 nm. The AgNPs-LTF gel with biologically processed AgNPs demonstrated effective infection control and enhanced wound healing outcomes. In Sprague–Dawley rats, the 4 mM AgNPs-LTF gel demonstrated significant wound closure, achieving complete closure by day 10, exceeding the healing rates of both the LTF and control groups. The AgNPs-LTF complex demonstrated high robustness and exceeded the performance of native LTF, exhibiting similar toxicity levels to AgNPs. The study shows the effectiveness of AgNPs-LTF gel in wound treatment, indicating its potential as a viable treatment option.

## Introduction

The management of wounds is a critical concern in healthcare due to the increasing prevalence of severe injuries, chronic wounds such as diabetic ulcers, and aging populations with slower healing abilities^1,2^. Common treatments involve gauze, cotton wool, and dressings to maintain moisture levels, manage exudates, and prevent infections^3,4^. However, these methods are limited in treating substantial injuries and persistent wounds^5,6^. To enhance wound healing, research has focused on incorporating biologically active substances and developing advanced dressings that can monitor physiological indicators like local stresses, temperature, and pH^7,8^. Despite progress, these advanced designs often involve complex fabrication processes, high costs, drug side effects, and challenges in loading and releasing bioactive chemicals.

Treating wound infections often necessitates the use of antibiotics; however, prolonged infections have resulted in the emergence of antibiotic-resistant microorganisms. More than 70% of infections are caused by bacteria resistant to commonly used antibiotics. As a result, recent research has focused on developing metal-based nanoparticles, chitosan, and its modifications, which demonstrate antibacterial properties, as alternative solutions^9–13^.

Metallic nanoparticles (MNPs), particularly silver^14^ and gold^15^, are widely recognized for their superior antibacterial properties compared to their ionic counterparts. Notably, no bacteria have demonstrated complete resistance to silver nanoparticles (AgNPs) so far^16^, underscoring the significant advantages of AgNPs utilization^17^. Despite MNPs’ adverse effects on organisms, their use is justifiable in palliative wound healing to alleviate patient suffering^18^. Moreover, MNPs can act as carriers for pharmaceutical drugs, showing synergistic effects when combined with other antibacterial treatments^19^. Studies have revealed that AgNPs functionalized with ampicillin exhibit a lower minimum inhibitory concentration (MIC) than free AgNPs and ampicillin alone, indicating the potential to achieve favorable outcomes with reduced AgNPs dosage^20^. This approach offers a way to mitigate medication-induced adverse effects. Additionally, biologically synthesized AgNPs, using methods involving probiotic bacteria, proteins, plant extracts, and similar approaches, have been documented to exhibit decreased toxicity^17^.

Previous research has extensively explored the intricate interactions between Ag^**+**^ ions and proteins^21–23^. According to the Lewis hypothesis, Ag + is classified as a soft acid, which readily binds with soft bases, particularly those containing sulfur groups. This understanding is invaluable for predicting metal-binding within protein sites. Covalent interactions between soft-acid metals and soft bases have been well-documented, including the rapid cleavage of disulfide bridges and the formation of chemical bonds with proteins via cysteine residues^23^. Molecular modeling techniques have been instrumental in investigating the complex interplay between Ag^**+**^ ions and bovine lactoferrin (LTF)^24^, highlighting the significant role of glutamic and aspartic acids in Ag^**+**^ binding by proteins. Notably, Ag^**+**^ demonstrates a higher affinity for N-containing functional groups than O-containing functional groups^25^. Additionally, it is important to note that silver compounds often exhibit photosensitivity, undergoing reduction to form metallic silver. Although silver nitrate, a commonly used Ag + salt, does not show photosensitivity, the interaction between Ag + ions and proteins often results in AgNPs forming^25,26^. These insights underscore the multifaceted nature of Ag^**+**^ interactions within biological systems.

Lactoferrin (LTF), a protein belonging to the transferrin family, binds non-heme iron and is vital for Fe^3+^ ion transportation and regulation. In addition to its immunological functions, LTF exhibits antibacterial^27^, antiviral^28^, antifungal^29^, immunoregulatory, and anti-inflammatory properties^30^. Recent studies highlight its role in promoting fibroblast and keratinocyte proliferation and migration, which is essential for wound healing. LTF accelerates wound closure in mice by modulating inflammatory responses and regulating fibroblast and keratinocyte activities^31,32^. Moreover, it contributes to the granulation phase by balancing fibroblast functions, including synthesizing hyaluronic acid and collagen breakdown.

LTF applied topically enhances wound closure^33,34^. Its antibacterial properties are attributed to several mechanisms: competing with lipopolysaccharide Ca2 + -binding sites on bacterial membranes, chelating Ca2 + through sialic acid in its glycans, hindering pathogen-eukaryotic cell interactions via high-mannose structures, and its highly cationic nature. Pepsin-mediated hydrolysis releases lactoferricin, which disrupts bacterial membranes when bound, further enhancing its antibacterial effect^35–39^.

Given the above facts, using LTF for synthesizing antimicrobial protein-based metallo composites appears appealing. It was reported that the synthesis of Ag-LTF nanocomposite using a reagent known as "Tollens’ reagent." This reagent combines silver with ammonia, specifically [Ag(NH_3_)_2_]^**+**^. Applying silver complexation with ammonia facilitates the dissolution of silver under alkaline circumstances while introducing considerable complexity to the chemical reactions involved in the process. Hence, exploring the potential interaction between LTF and Ag^**+**^ under acidic circumstances is intriguing. Furthermore, it is imperative to investigate the effects of varying concentrations of Ag^**+**^ on the characteristics of the corresponding Ag-LTF complexes^40^. In another approach, AgNPs were synthesized by utilizing Spent mushroom substrate (SMS) as a reducing agent and chitosan as a stabilizing agent, together with the incorporation of GO or LTF to produce AgGO and Ag-LTF, respectively^41^. The finding reveals that AgNPs, Ag-LTF, and AgGO have antibacterial properties, potentially serving as alternative antibacterial agents.

This work aims to create AgNPs by utilizing LTF to produce AgNPs-LTF with varying silver concentrations (1, 2, and 4 mM). Carbopol was used to convert all concentrations of AgNPs-LTF into a gel form. Subsequently, their biological activity—specifically regarding antibacterial effectiveness, antioxidant potential, and in vivo wound healing capability—was evaluated. AgNPs were characterized using various analytical techniques, including UV–Vis spectroscopy, X-ray diffraction (XRD), Fourier Transform Infrared spectroscopy (FT-IR), zeta potential analysis, scanning electron microscopy (SEM), energy-dispersive X-ray spectroscopy (EDX), and transmission electron microscopy (TEM).

## Experimental

### Chemicals

The chemicals and supplies used in this study were sourced from Sigma-Aldrich, located in Steinheim, Germany. These included bovine whey from bovine milk, sodium chloride, sodium hydroxide, phosphate-buffered saline (PBS), Carbopol, and silver nitrate with a 99.99% trace metals base purity.

### Lactoferrin isolation

Lactoferrin (LTF) was extracted from bovine whey, following the methodology established in a prior study with minimal modifications^21^. Initially, whey was diluted in a 1:1 ratio with a 25 mM phosphoric buffer containing 0.3 M NaCl. Subsequently, the specimen underwent defatting through centrifugation at 8000 rpm for 20 min at 25°C. The resulting transparent liquid was then transferred to glass chromatographic columns filled with SP Sepharose TM fast flow and subjected to separation using a linear NaCl gradient (0.2 to 0.8M) in a 30 mM phosphoric buffer. The target fraction was monitored at 280 nm and underwent centrifugation at 4000 rpm and 25 °C, employing Amicon Ultra-15 Centrifugal Filter Units with a 30 kDa molecular weight cut-off. The reddish-colored protein sample collected from this process underwent lyophilization and was subsequently stored at − 20 °C.

### AgNPs synthesis using lactoferrin

**Fig. 1:**
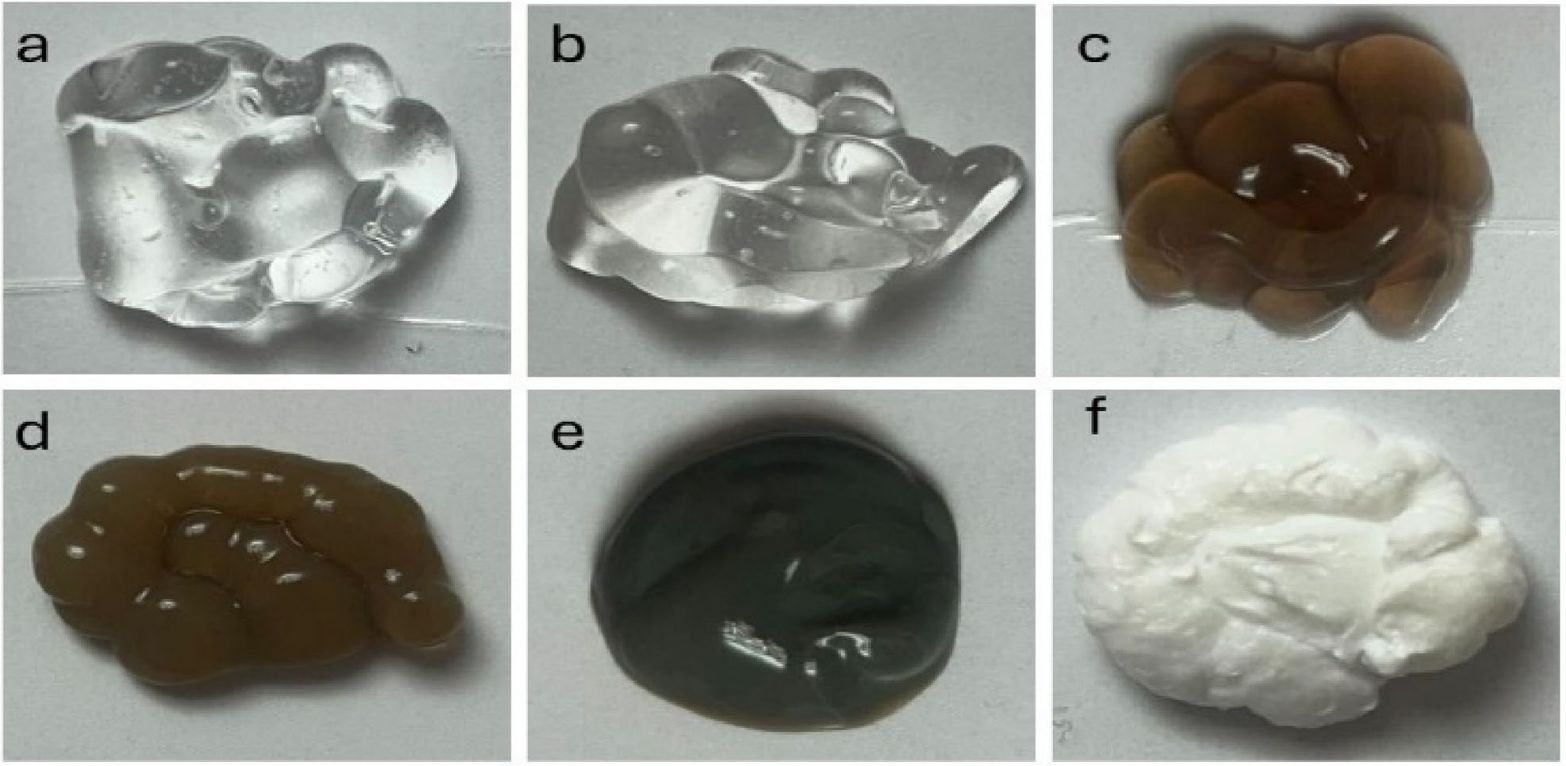
real images of the prepared gel samples. control placebo gel (**a**), LFT gel (**b**), 1mM AgNPs-LTF gel (**c**), 2mM AgNPs-LTF gel (**d**), 4mM AgNPs-LTF gel, and Silver sulfadiazine gel (**e**).

A precise volume of 10 mL of LTF extract was meticulously introduced into an aqueous solution containing 1, 2, and 4 mM of AgNO_3_ in H_2_O (40 mL). The mixture was placed in a dark environment and subjected to magnetic stirring at room temperature for one hour. A noticeable color change from a pale yellow hue to a deep brown shade indicated the formation of silver nanoparticles (AgNPs). The entire solution was stirred for 6 h to ensure complete reduction. Following this, a portion of the solution underwent centrifugation at 15,000 rpm for 30 min, and the upper part was separated by decantation. The obtained AgNPs were washed with a water–ethanol mixture (1:3 ratio) to eliminate any uncoordinated biological substances. The resulting powder underwent drying in an oven at 50 °C for two hours to prepare for subsequent characterization procedures. To each solution, including three different concentrations of silver, 2 g of Carbopol powder was added. The pH of the liquids was then adjusted to an alkaline condition using a 1 mM NaOH solution. This process yielded the development of a gel composed of AgNPs-LTF, tailored explicitly for use in biological contexts (Fig. 1).

### Characterization

The absorbance peak of AgNPs-LTF was determined using a UV–Vis spectrophotometer (Bio Aquarius CE 7250, UK) with a scanning range of 200 to 800 nm. The Ag colloids were also verified using a double-beam auto fluorescence spectrophotometer. The particle size distribution of AgNPs was investigated using dynamic light scattering (DLS) with the nanoparticle SZ-100V2 Series instrument. The charge of AgNPs-LTF was determined using the Brookhaven ZetaPALS instrument, specifically by measuring the zeta potential. The particle size of AgNPs-LTF was examined using Japan’s JEOL 2100 electron microscope. High-resolution transmission electron microscopy (TEM) images were obtained at an acceleration voltage of 200 kV. The FE-SEM instrument, specifically the QUANTA-FEG250 model from the Netherlands, was employed to analyze and characterize the morphology of the produced sample. The structural investigation was conducted using XRD pattern analysis. The XRD measurements were performed using a Pertpro diffractometer from the USA, with Cu Kα1 radiation having a wavelength of 1.5404 Å, operating at 45 kV and 40 mA.

### Antibacterial activity

The antibacterial effectiveness of AgNPs-LTF gel with concentrations of (1, 2,4 Mm) was evaluated using a zone of inhibition method, following established protocols with minor modifications, against Gram-positive *Staphylococcus aureus* (*S. aureus*) and Gram-negative *Escherichia coli* (*E. coli*) bacteria^42,43^. A volume of 100 μL of diluted bacterial suspensions, with an approximate concentration of (10^6^ CFU mL^−1^), was carefully transferred and evenly spread over a nutrient agar culture medium. After a two-hour sterilization process using UV irradiation, a gel containing AgNPs-LTF, weighing 0.1 g, was applied over the culture medium, and a control antibiotic, Ciprofloxacin, in disc form, was included for comparison. After a 48-h incubation period at 37 °C, the average diameters of inhibition zones were measured at three randomly selected positions within the zones.

### The antioxidant potency of AgNPs-LTF

The antioxidant capabilities of AgNPs-LTF were evaluated by subjecting them to stable DPPH, following established procedures documented in prior publications^44^. Vitamin C was employed as the standard for comparison. Various concentrations of AgNPs-LTF, ranging from 0.25 to 2.0 mg/ml, were mixed with 0.5 mL of DPPH (1 mM) solution dissolved in methanol. The mixture was then incubated in the dark at room temperature. After the incubation period, the UV absorbance of the samples was measured at a wavelength of 517 nm, with methanol serving as the reference. The negative control included a DPPH solution and methanol without AgNPs-LTF. The experiment was conducted in three separate sets, and the percentage of scavenging activity of AgNPs-LTF was calculated using the provided formula:

1 $${\text{Inhibition }}\;{\text{percentage}}\;{\text{of}}\;{\text{ DPPH }}\left( {\text{\% }} \right) = \frac{{{\text{Abs}}.{\text{ control}} - {\text{Abs}}.{\text{ sample }}}}{{{\text{Abs}}.{\text{ control}}}}{ } \times { }100{ }$$

### Anti-inflammatory properties in vitro

The in vitro assessment of the anti-inflammatory effectiveness of LTF and AgNPs-LTF gel complexes were conducted using the protein denaturation method described in prior work, with minor adjustments^45^. The study utilized Diclofenac sodium, a potent nonsteroidal anti-inflammatory drug, as a control. In the experimental setup, 100 mg of LTF and AgNPs-LTF gel were introduced into a solution of 2.0 mL of PBS with a pH 6.4. Additionally, 2 mL of albumin derived from recently laid eggs at a concentration of 1 mM was included in the combination. The resulting mixture was then subjected to an incubation period of 20 min at 37 °C. The denaturation process was accomplished by subjecting the solution to thermal treatment in a water bath maintained at a temperature of 60 °C for 15 min. The optical density measurement was conducted at a wavelength of 660 nm under normal ambient temperature conditions. The experiment was conducted in triplicate. The equation utilized in this study was to assess the extent of inhibition of protein denaturation, expressed as a percentage:

2 $${\text{Denaturation }}\;{\text{Inhibition }}\left( {\text{\% }} \right) = { }\frac{{{\text{ODc }} - {\text{ ODs}}}}{{{\text{ODc}}}}{ } \times { }100$$

ODs = Optical density of test sample.

ODc = Optical density of control.

### In vivo wound healing of AgNPs-LTF

Rats were acquired from the faculty’s animal house, and approximately one cm diameter circular incisions were made on the dorsal surface, designating wound models. The rats were randomly allocated into 6 groups (3 rats per group). The wounds were treated using a gel of 200 mg/day. The treatment was as follows: Group (I) a control placebo gel; Group (II) a gel containing AgNPs-LTF (1mM); Group (III) a gel containing AgNPs-LTF (2mM), Group (IV) a gel containing AgNPs-LTF (4Mm), Group (V) Gel containing LTF only, and Group (VI) silver sulfadiazine cream from the national market. Subsequently, the wounds were subjected to applying the gel mentioned above for 16 days. Photographs were taken, and dimensions of the wounds were recorded to assess the progression of wound healing. Wound area and healing percentage were recorded at designated time intervals, and the relative wound area was calculated using the provided equations:

3 $${\text{Wound }}\;{\text{area \% }} = \frac{{{\text{wound}}\;{\text{ area}}\;{\text{ at }}\;{\text{certain }}\;{\text{day}}}}{{{\text{wound}}\;{\text{ area}}\;{\text{ at}}\;{\text{ day }}\;0}} \times 100$$

### Ethical statement

Male rats, aged 8 months, were randomly allocated into six experimental groups, with three rats per group (n = 3/group). All experimental procedures were reviewed and approved by the Institutional Animal Care and Use Committee (IACUC) of the Faculty of Pharmacy, Damanhur University, ensuring compliance with national and institutional guidelines for the care and use of laboratory animals. The study was conducted under approval number 318PT6.

Euthanasia was performed humanely at the end of the experimental procedures. The rats were euthanized using **an overdose of sodium pentobarbital (150 mg/kg, intraperitoneal injection)**; this study was conducted and is reported following the ARRIVE guidelines to ensure transparency, reproducibility, and ethical rigor in animal research. All necessary steps were taken to minimize animal suffering, and ethical standards were followed throughout the study.

### Statistical evaluation

The studies were performed on three successive occasions, and the outcomes were reported as the mean value and the accompanying standard deviation. The statistical analysis was conducted using the SPSS software. A t-test was performed, resulting in the acquisition of a *P*-value. Results *P* > 0.05 non-significant, *P* < 0.05: significant, *P* < 0.001: highly significant.

## Results and discussion

**Fig. 2:**
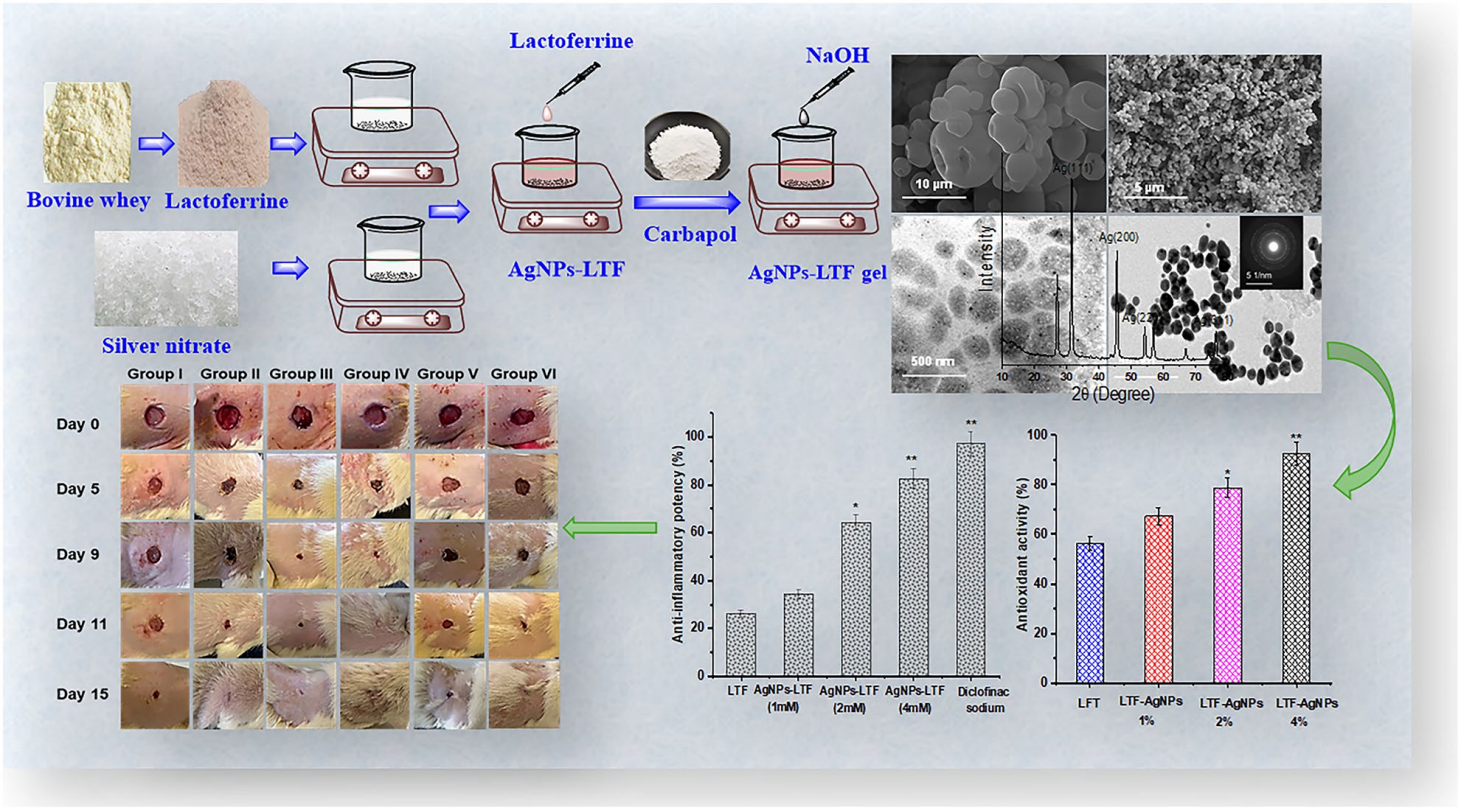
Green synthesis process of lactoferrin-infused AgNPs and their potential applications.

Figure 2 illustrates that the green synthesis of lactoferrin-infused AgNPs gel offers a sustainable and innovative approach to wound healing. By leveraging the natural bioactivity of lactoferrin and the antimicrobial properties of silver nanoparticles, this biocompatible gel addresses critical challenges in wound management, including infection control and tissue regeneration. The environmentally friendly synthesis process eliminates the need for harmful chemicals, ensuring a safer and more sustainable production method. Initial evaluations demonstrate that the lactoferrin-AgNPs gel promotes cell proliferation, accelerates wound closure, and maintains structural stability and biocompatibility, making it a promising candidate for advanced wound care applications.

### Optical properties and geometry optimization

**Fig. 3:**
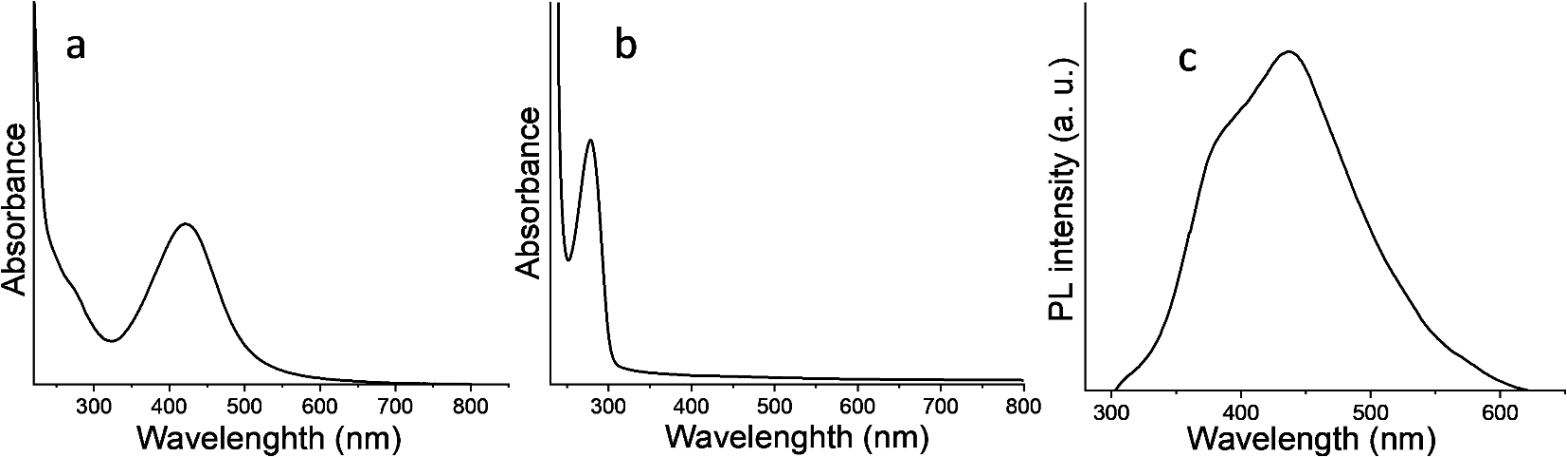
Spectrophotometry of (**a**) AgNPs, (**b**) LTF [λ = 220–800 nm], and (**c**) PL of AgNPs-LTF.

UV–visible spectroscopy is a prevalent silver nanoparticle (AgNPs) characterization method, providing valuable insights. The recorded peak at a specific wavelength serves to confirm nanoparticle production, establishing a correlation with their average diameter. The vibrant coloration of AgNPs arises from the collective oscillation of conduction electrons induced by an electromagnetic field, a phenomenon characterized by the surface plasmon resonance (SPR) absorption feature. Figure 3a shows the stable SPR band at 427 nm for Ag colloids in the UV–visible range. The interband transition at approximately 245 nm contributes to a broad UV spectrum. The asymmetric SPR band indicates the presence of spherical nanoparticles, while the elongated tail in the red region signifies the polydisperse nature of nanoparticle sizes.

The photoluminescence (PL) of AgNPs is greatly influenced by the electrical interactions between the upper d-band and the conduction Sp-band., as depicted in Fig. 3b. By employing an excitation wavelength of 380 nm, the PL spectra of AgNPs exhibit a conspicuous and well-defined peak centered at about 450 nm, exhibiting a vigorous intensity (Fig. 3c). This observation implies an enhancement in nanoparticle Synthesis, consistent with prior research^46^. The occurrence in question can be attributed to the activation of electrons in d-orbitals, leading to their transfer to the higher Fermi level. The relaxation process of electrons in the Sp-band occurs due to electron–phonon scattering, leading to energy transfer to holes in the d-band. AgNPs’ luminescence relies upon this phenomenon, rendering them sensitive to visible light.

### FT-IR

**Fig. 4:**
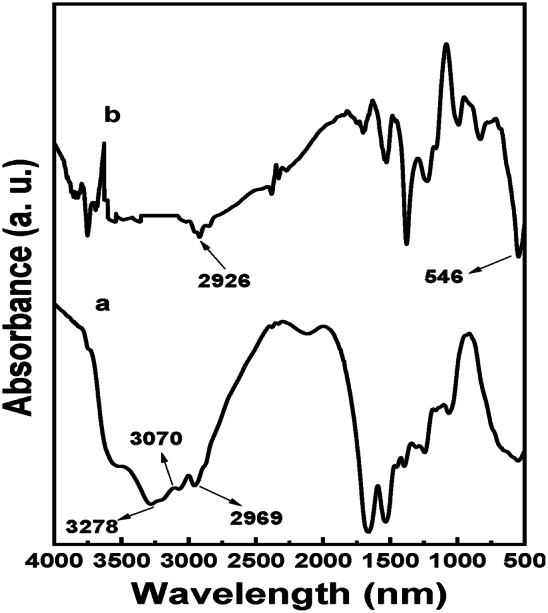
FTIR spectra of (**a**) LTF and (**b**) AgNPs-LTF [4000–500 cm^−1^].

FT-IR is a scientific tool that enables monitoring alterations in the absorbance of certain functional groups during protein interactions^22^. Figure 4 displays the absorption bands obtained from the native LTF and the formed AgNPs-LTF complexes. The native LTF spectrum has characteristic absorption bands that are exclusive to proteins. The 2800–3700 cm^−1^ spectral range corresponds to the vibrational modes of functional groups containing hydrogen atoms. The broad spectral peak centered at 3278 cm^−1^ corresponds to the vibrational modes of O–H and N–H groups, which actively engage in hydrogen bonding interactions. The peak observed at 3078 cm^-1^ corresponds to C-H bonds within aromatic rings. Additionally, spectral peaks falling within 2750–3000 cm^−1^ can be assigned to doublets corresponding to the symmetric and asymmetric vibrations of C-H bonds in methyl and methylene groups^47^. Observing shifts and variations in signal intensity levels following the interaction between LTF and Ag^+^ suggests structural changes in the protein. The integration of AgNPs into proteins is expected to elicit certain alterations. For example, the vibrational band seen at a wave number 2969 cm^−1^ in the LTF moved to 2926 cm^−1^ in the AgNPs-LTF complex. The stretching frequency for AgNPs formation was determined around 546 cm^-1^. The presence of additional bands for the respective groups can also be observed: δ as(CH_3_) at 1462 cm^−1^ and δ (CH_2_) at 1441 cm^−1^^47,48^. As reported in a previous study, the glycoprotein as LTF exhibits a glycan content that varies between 6.7% and 11.1% of its overall molecular weight^49^. Therefore, the spectral bands with wave numbers below 950 cm^−1^ can be attributed to the vibrations of v(C-O), νst(C-O), and νst(C–C) in glycans^50^. Hence, the alterations seen in the specific area can be attributed to either the interaction with the side chains of the corresponding amino acids or the ingredients of protein glycans.

### XRD

**Fig. 5:**
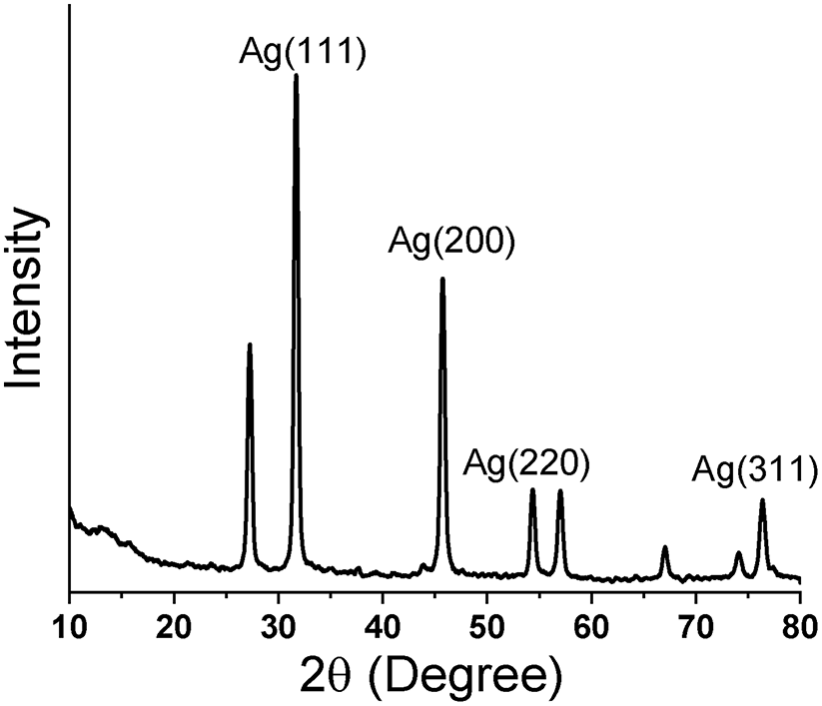
XRD identification of LTD-AgNPs.

The XRD investigation confirmed the crystalline structure of the AgNPs, as depicted in Fig. 5. The presence of sharp and distinct peaks in the peak intensity suggests a significant level of crystallinity in the AgNPs. The presence of diffraction peaks at 32.7°(111), 45.5°(200), 54.2°(220), and 65.40°(311) in the 2θ can potentially be ascribed to the reflection planes of the face-centered cubic structure of metallic AgNPs, as documented in the JCPDS database with the number 04–0783 in the year 1991. The results of our study align with the contemporary methodology, wherein the intricate biomolecules present in the extract of *V. vinifera fruit peel* were employed to manufacture AgNPs. This biosynthesis yielded nanocrystals that exhibited identical characteristics, as confirmed by XRD examination^51,52^. The Debye–Scherrer equation estimates the average size of crystallites or grains.

4 $${\text{D }} = {\text{ k}}\lambda /\beta {\text{ cos }}\theta$$

The average crystal size is denoted by D, the Scherrer coefficient is represented by k (with a value of 0.9), λ signifies the X-ray wavelength, β corresponds to the full-width half-maximum intensity, and θ denotes the diffraction angle. The mean size of the AgNPs crystallites was determined to be 16.4 nm.

### Morphological characterization of synthesized AgNPs

The synthesis of metal nanoparticles (MNPs) involves a complex, multi-step process encompassing nucleation, growth, and aggregation stages^53^. Key factors impacting the size and distribution of these nanoparticles at each stage include the metal concentration and the quantity of the capping agent involved. Specifically, lactoferrin (LTF) assumes the dual role of a reducing and capping agent in this process. Its function involves the reduction of free Ag^**+**^ ions to Ag^**0**^, leading to the formation of a complex known as AgNPs-LTF, thereby facilitating the synthesis of AgNPs.

Determining the zeta potential (ZP) provides crucial insight into the electric charge present on nanoparticles within a specific medium Table 1. Typically, multiple charges on nanoparticle surfaces result in repulsive interactions. In this study, lactoferrin (LTF) was utilized in the synthesis of silver nanoparticles (AgNPs), resulting in a measured ZP value of + 19.6 mV for LTF and − 21.7 mV for AgNPs-LTF, indicating a negative charge on the AgNPs’ surface. This negative charge effectively prevented nanoparticle aggregation in water, facilitating the formation of stable AgNPs. The stability of the synthesized AgNPs dispersed in water was exceptional, as evidenced by the obtained results, meeting the requisite stabilizing charge threshold of either + 30 mV or 30 mV for sustained stability^54^.

**Table 1:** Zeta potential analysis of LTF and AgNPs-LTF.

Sample	Zeta potential	Mobility µm/S/V/cm	Conductivity µs/cm	Run time	Viscosity
LTF	+ 19.6	1.53	152	60 s	0.876
AgNPs-LTF	− 21.7	1.7	242	60 s	0.867

**Fig. 6:**
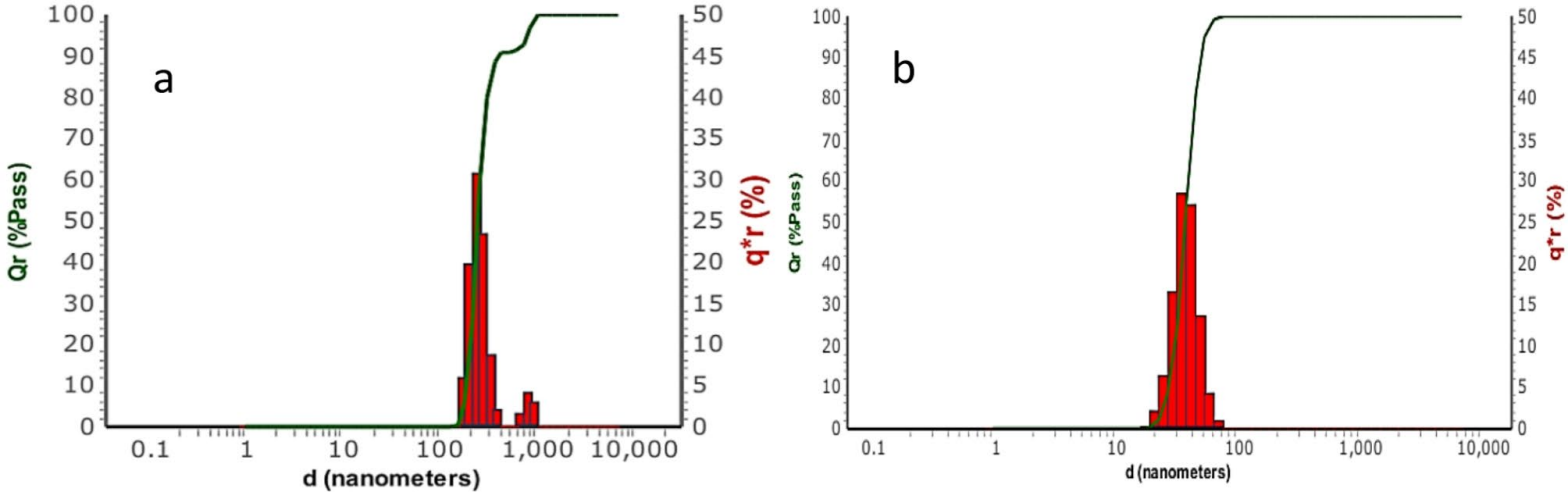
Particle size distribution of (**a**) LTF and (**b**) AgNPs-LTF.

Dynamic light scattering (DLS) is a reliable method to gauge the particle size distribution within nano-scale materials in colloidal suspensions or solutions^55^. The size distribution analysis of both precursor LTF and AgNPs-LTF is presented in Fig. 6. While the DLS for precursor LTF measured 228.2 nm, the examination revealed that the AgNPs demonstrated polydispersity, showcasing a mean particle size of 25.81 ± 2.31 nm. The final dimensions of these nanoparticles rely on the biomaterial adsorbed onto the AgNPs and the characteristics of the electrical double-layer within the solvent surrounding them^56^. Notably, AgNPs exhibit a monodispersed nature, which is evident from their low polydispersity index (PDI) value of 0.11.

**Fig. 7:**
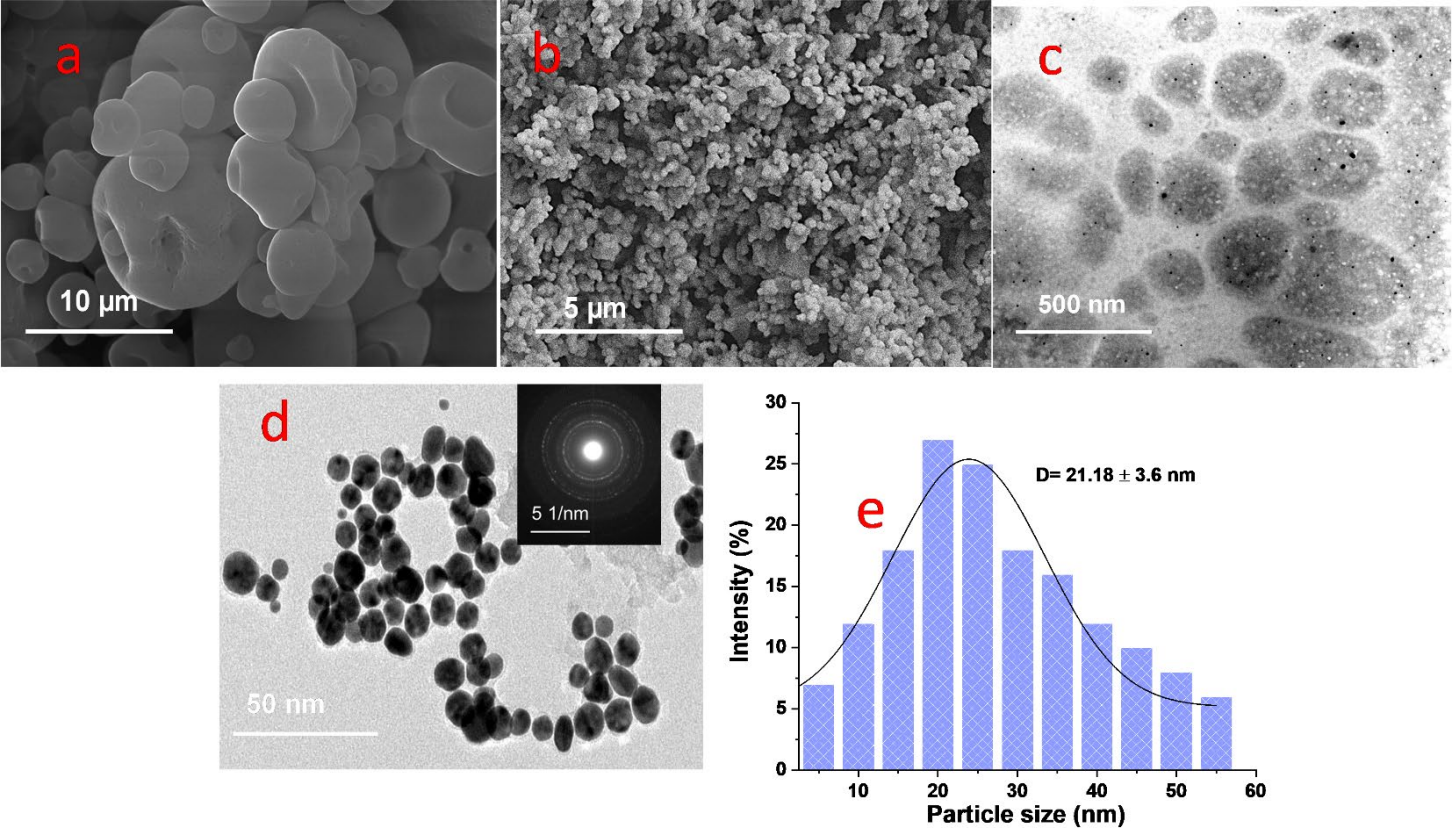
SEM texture of (**a**) LTF, (**b**) AgNPs-LTF, TEM microstructural of (**c**) LTF, (**d**) green synthesized AgNPs by LTF (inset SAED of AgNPs), and (**e**) TEM histogram of AgNPs by LTF.

The SEM analysis was utilized to scrutinize the morphological characteristics of the complexes resulting from the interaction between AgNPs and LTF, as displayed in Fig. 7. The representative images illustrate spherical structures in both cases, signifying the successful creation of hybrid nano-bio carriers via a nanoprecipitation technique. The surface texture of LTF exhibits a buckled shape and hollow interior (Fig. 7a), which is a relatively larger size. Upon the addition of LTF to silver salt, there’s a noticeable size reduction (Fig. 7b). These findings suggest that the lengthy glycoprotein chain could adopt a more compact form, leading to a uniform alignment of the protein surrounding the silver and the proper shape^57^. The SEM imagery of the developed nanoparticles aligns with prior studies on different hybrid nanoparticles^31^. The microstructural analysis conducted via TEM revealed individual spherical entities of AgNPs-LTF, demonstrating a homogeneous distribution, spherical shape, and irregular surface particles with well-defined sizes as referred in Fig. 7c,d with a detected tiny particle size ~ 25 nm. The Selected Area Electron Diffraction (SAED) analysis results were consistent with those of XRD. The characteristic diffraction rings indexed as (111), (200), (220), and (311) align with the fcc lattice structure typical for AgNPs. These findings concur with previous studies^58–60^. They are clearly distinguishing the structure of the nanoparticles from the nanocomposites. Researchers have posited that smaller particles might exhibit enhanced antimicrobial activity, given their potential to more effectively penetrate bacterial cells, disrupting their cell walls^61^. Figure 7e depicts the TEM histogram of AgNPs/LTF demonstrating an average diameter of 21.18 ± 3.6 nm. This result shows good agreement with the particle size distribution data, which indicated an average size of 25.81 ± 2.31 nm. The close correlation between these values highlights the reliability of the characterization methods used and confirms the uniformity of the synthesized AgNPs.

### Antibacterial potency

Human wound fluids serve as a breeding ground for pathogenic bacteria, fostering infections that undermine the efficacy of wound dressings^62,63^. To expedite wound healing, it becomes crucial to administer antimicrobial agents capable of eradicating these harmful bacteria. Paradoxically, a conducive medium for bacterial growth should also be provided to evaluate the effectiveness of these antimicrobial agents. Gel formulations emerge as promising candidates for such applications because they assimilate antibacterial agents like AgNPs. Moreover, gels enable controlled in vitro release of these agents into wounds, amplifying their therapeutic effectiveness.

**Fig. 8:**
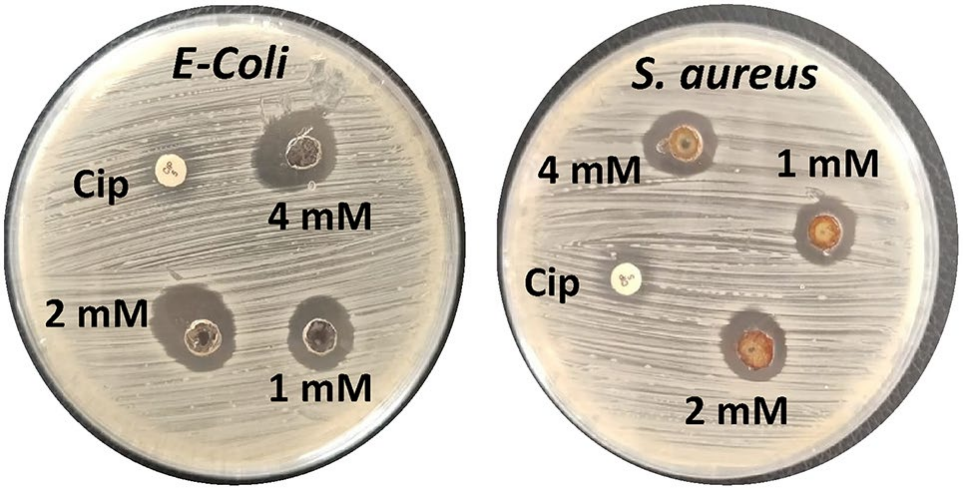
Zone of inhibition of AgNPs-LTF gel (1, 2, 4mM) against *E. coli* and *S. aureus* [n = 3].

**Fig. 9:**
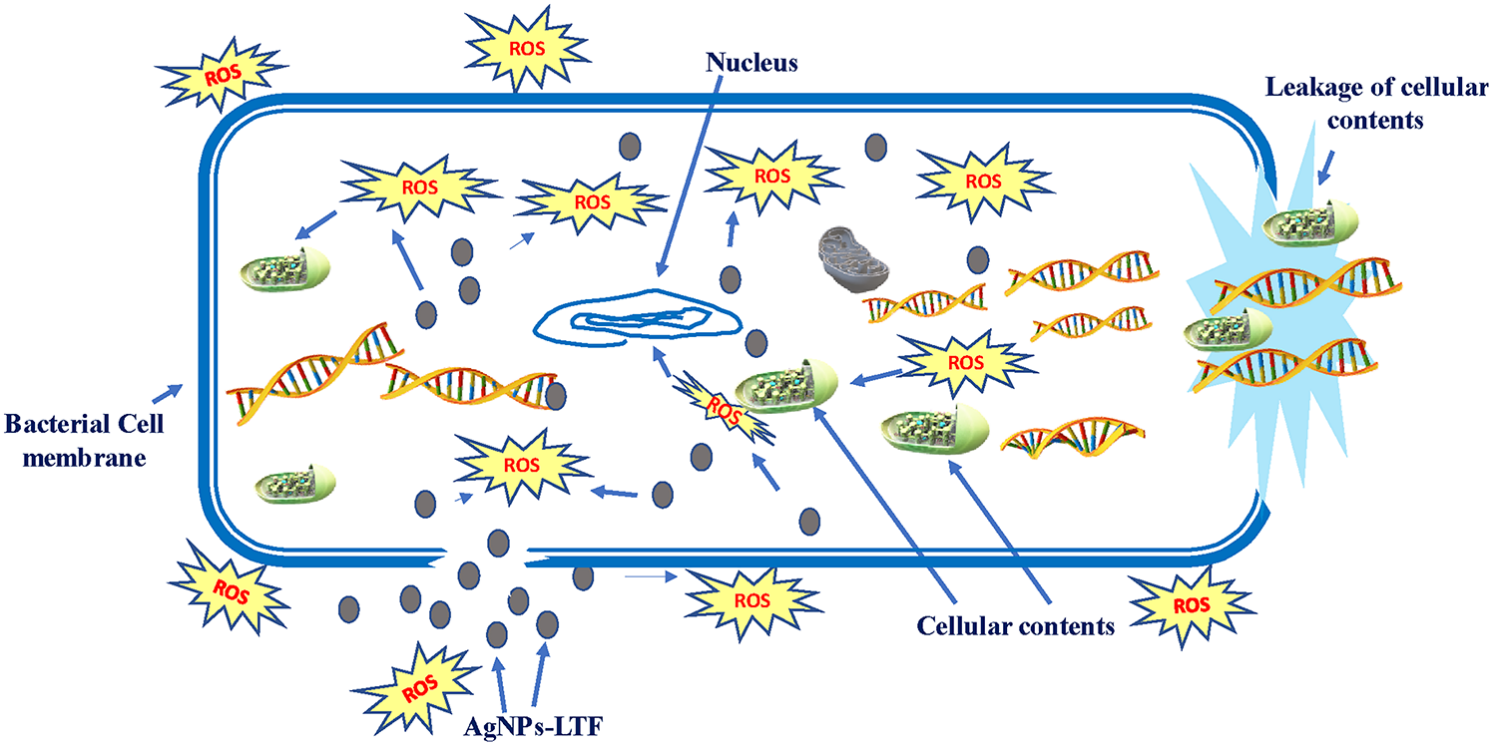
The inhibition of bacterial growth proposed mechanism.

The antibacterial efficacy of AgNPs-LTF gel in different concentrations of silver (1, 2, and 4 mM) was assessed using the ZOI method. The bacteria used for this study were *E. coli* and *S. aureus*. Figure 8 illustrates the results of the ZOI tests at different concentrations. In the presence of AgNPs-LTF at (4 mM), the ZOI exhibited a larger diameter of 26.2 ± 2.5 mm and 24.3 ± 3.2 mm against *E. coli* and *S. aureus*, respectively. Similarly, the ZOI diameter for AgNPs-LTF (1 mM and 2 mM) were measured at (19.1 ± 1.3 mm, 17.1 ± 2.1 mm) and (23.1 ± 1.3 mm, 22.1 ± 1.5 mm) against *E. coli* and *S. aureus*, respectively. Notably, these values were more significant than the ZOI exhibited by the standard antibiotic ciprofloxacin (10µg), measured at (17.1 ± 2.3 mm, 16.1 ± 3.2 mm). This observation demonstrates notable antibacterial efficacy against both species and a substantial AgNPs release from the gel formulation. Furthermore, this finding suggests a positive correlation between the concentration of AgNPs and the level of antibacterial activity. Based on the illustration of the mechanism underlying bacterial death caused by AgNPs in Fig. 9, it can be inferred that the antimicrobial efficacy of the AgNPs-LTF gel is mainly attributed to the existence of AgNPs. This observation indicates that AgNPs can adhere to and penetrate the bacterial cell wall. The cell’s structural integrity is compromised due to this phenomenon. Furthermore, AgNPs’ generating free radicals is often considered a crucial determinant in cellular demise. Moreover, the thiol groups present in numerous vital enzymes can form complexes with silver ions, thus leading to their loss of activity^64,65^. Moreover, the interaction between AgNPs and DNA disrupts DNA replication, ultimately leading to bacterial cell death.

In contrast, *E. coli* exhibited a comparatively more significant antibacterial effect than *S. aureus*, mainly attributable to variances in the membrane structure of the two bacterial species. The observed impact of AgNPs on *E. coli* was more pronounced due to its comparatively thinner cell wall than *S. aureus*^66^. Finally, the findings indicated that the AgNPs-LTF gel, in its original state, exhibited efficacy as a medium for combating bacterial growth.

### Antioxidant activity

**Fig. 10:**
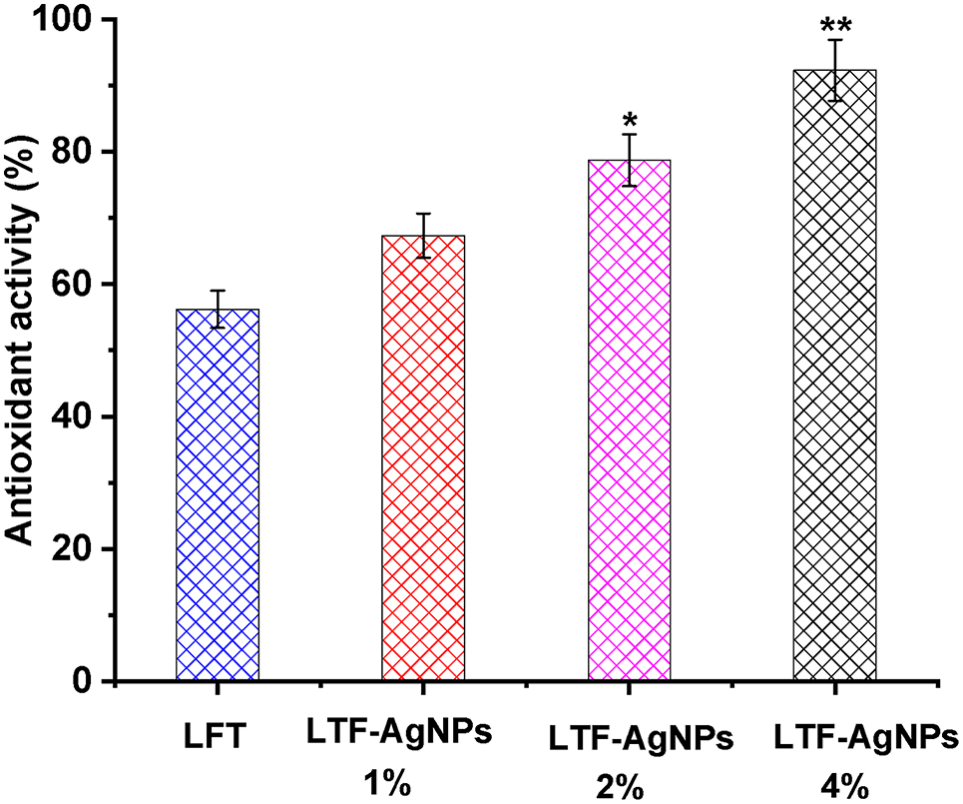
Antioxidant potency of AgNPs-LTF gel (1, 2, 4mM) against DPPH (*): *P* < 0.05: significant, (**) : *P* < 0.001: highly significant.

In the context of skin injury, during the inflammatory phase of wound healing, a significant amount of reactive oxygen species (ROS) is generated, leading to cellular damage characterized by the degradation of lipids, proteins, and nucleic acids. Consequently, this cellular injury disrupts the wound-healing process’s progression, resulting in cell death. Antioxidants have been found to have a substantial impact on enzyme repair processes and metabolic function enhancement^67^. A multitude of research has provided evidence supporting the reliability of nanoparticles as a viable source of antioxidant activity^68,69^. The current investigation sought to evaluate the effectiveness of LTF and AgNPs-LTF gels in eliminating free radicals. These gels incorporate silver at 1, 2, and 4 mM concentrations. A DPPH-free radical scavenging experiment was conducted for this assessment, and the results are presented in Fig. 10. The AgNPs-LTF (4 mM), exhibited the most influential radical scavenging activity against DPPH, with a percentage of 92.3%. The radical DPPH scavenging activity of LTF was found to be the lowest at 56.2%, consistent with the findings of a prior study with comparable results^70^. The presence of diverse compositions leads to a significant shift. In the present study, it was observed that the AgNPs-LTF gel exhibited a robust antioxidant capability. This observation suggests that the augmentation of AgNPs content enhances their power to transport more ions effectively. The accelerated wound healing and subsequent improvement in individual health growth can be attributed to the free radical collection approach employed for AgNPs-LTF gel. Moreover, one may contend that the regulation of composition yields substantial enhancements in biological behaviors contingent upon the type of components from a prospective standpoint.

### Anti-inflammatory properties

**Fig. 11:**
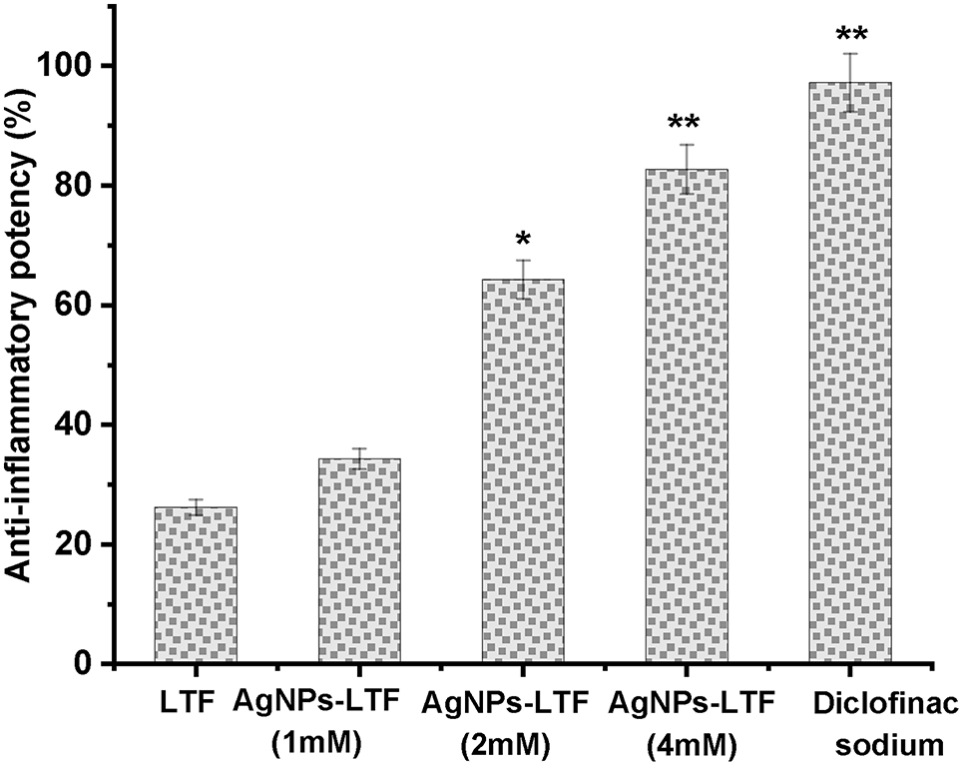
Anti-inflammatory potency of AgNPs-LTF gel (1, 2, 4mM) using the protein denaturation method. (*): *P* < 0.05: significant, (**) : *P* < 0.001: highly significant.

The process of wound dressing can be delineated into three distinct yet interconnected phases, including inflammation and tissue remodeling^71^. Therefore, the utilization of anti-inflammatory medications is crucial in expediting the process of wound healing. A protein denaturation assay was performed to evaluate the comparative anti-inflammatory efficacy of various AgNPs-LTF gel formulations against the standard drug Diclofenac sodium. The denaturation of proteins exhibited substantial variation (p < 0.001) among the different gel formulas containing AgNPs-LTF. The Diclofenac sodium that adheres to the usual protocol had the most pronounced anti-inflammatory activity, with a measured efficacy of 97.2 ± 1.6%. The AgNPs-LTF gel (4mM) demonstrated a lower than Diclofenac sodium but notable anti-inflammatory potential, with an efficacy of 82.7 ± 1.9%. The AgNPs-LTF gel (2mM) exhibited a further decrease in anti-inflammatory potency, measuring at 64.3 ± 3.3%. Finally, the AgNPs-LTF gel (1mM) displayed anti-inflammatory activity, with an efficacy of 34.3 ± 2.6% and the activity of LTF was found to be the lowest at 26.2% (Fig. 11)^72–74^. The data was collected in triplicate and presented as the mean ± standard deviation. Statistical analysis revealed substantial variances (*p* < 0.001) across the different formulations of produced nanofibers. This finding corroborates earlier findings indicating that utilizing an aqueous ethanolic bark extract of *Mangifera indica* for synthesizing AgNPs yields particles with remarkable anti-inflammatory properties. These nanoparticles exhibited significant inhibition (85%) against egg albumin denaturation, aligning with previous research in this domain^75^. In an alternative method, silver nanoparticles were produced utilizing *Brachychiton populneus* leaf extract in an aqueous medium. The inhibition of albumin denaturation exhibited a direct correlation with the concentration of AgNPs, with maximal inhibition reaching 81.13% at a concentration of 500 µg/mL^76^.

### In vivo wound healing

The evaluation of the healing potential for wounds was conducted using laboratory rats. The rats were subjected to wounds measuring approximately one cm in diameter on their dorsal region. The rats with wounds were haphazardly allocated into 6 groups, each consisting of 3 rats, according to the treatment approach.

**Fig. 12:**
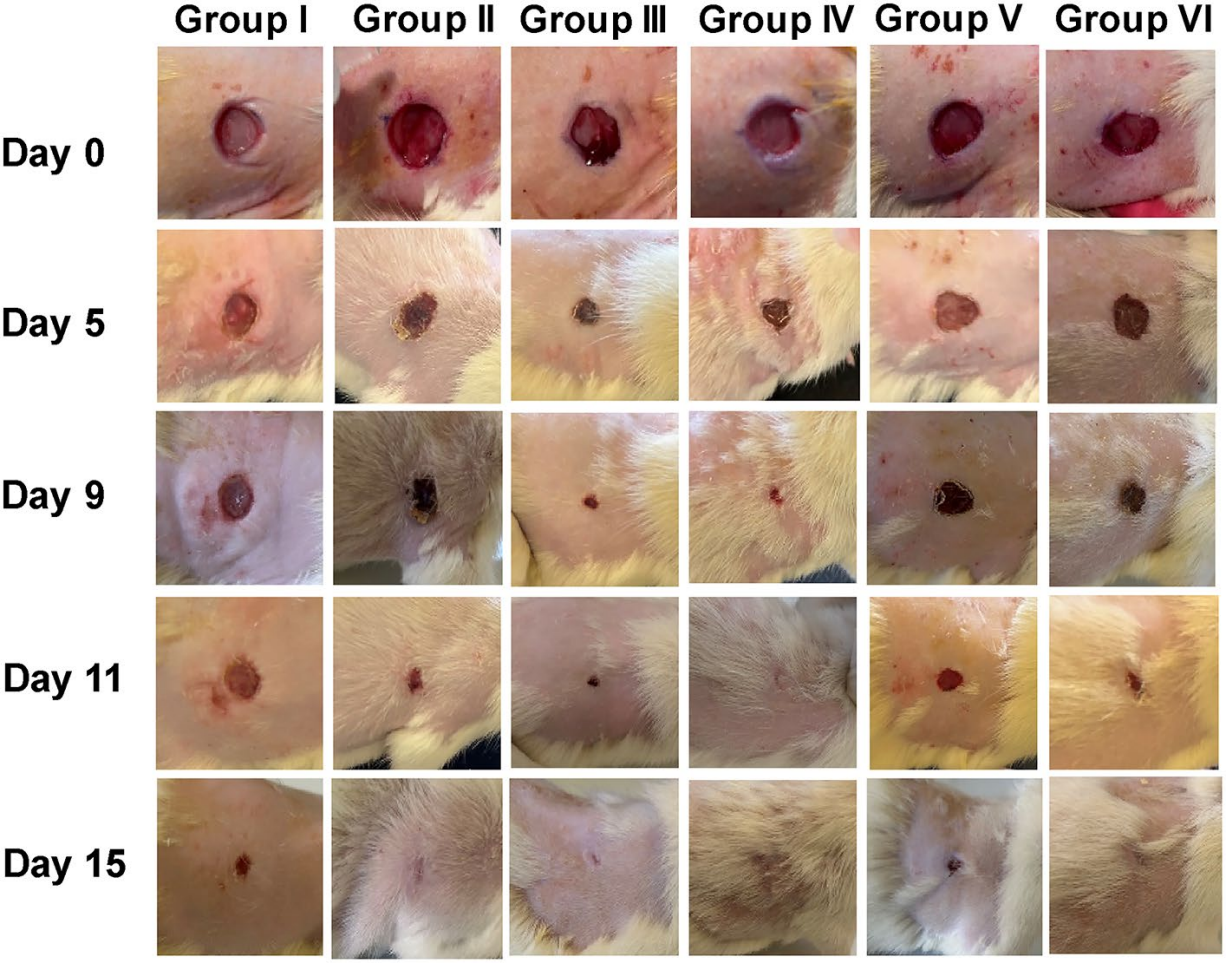
Progress of cutaneous wound healing in group (I) a control placebo gel, Group (II), a gel containing AgNPs-LTF (1 mM); Group (III) a gel containing AgNPs-LTF (2 mM); Group (IV) a gel containing AgNPs-LTF (4 Mm), Group (V) Gel containing LTF only and Group (VI) silver sulfadiazine cream from the national market.

**Fig. 13:**
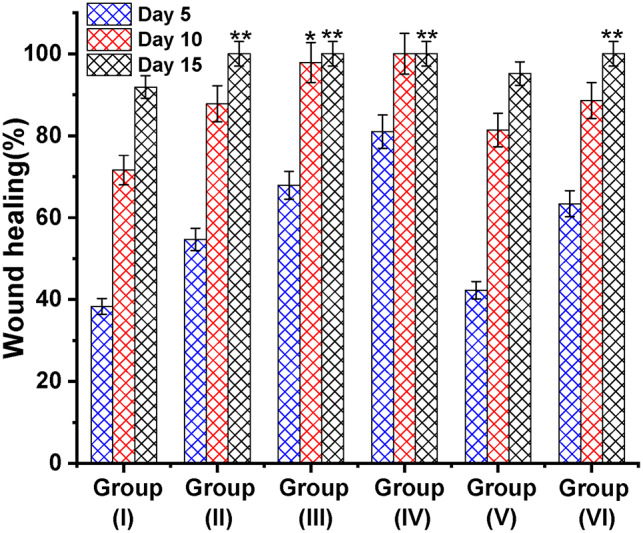
Average healing percentage for each group over time. Where Group (I) a control placebo gel, Group (II) a gel containing AgNPs-LTF (1 mm), Group (III) a gel containing AgNPs -LTF (2 mm), Group (IV) a gel containing AgNPs -LTF (4 Mm), Group (V) Gel containing LTF only and Group (VI) silver sulfadiazine cream from the national market. (*): *P* < 0.05: significant, (**) : *P* < 0.001: highly significant.

The group treated with AgNPs-LTF gel (4 mM) had the most rapid healing rate, as depicted in Fig. 12. The wounds exhibited closure on the 9th day and achieved complete healing on the 10thday. The process of hair creation was also noticed in the vicinity of the wound site; this indicates that the AgNPs-LTF gel (4 mM) had low cytotoxicity to skin tissue. This is compared to the LTF-treated group, which depicted behavior somewhat better than the control group. On the 10th day, the control group exhibited an average remaining wound area of 28.34%. In contrast to the group treated with AgNPs-LTF gel (4mM), the group treated with AgNPs-LTF gel (1 mM) exhibited a comparatively slower rate of wound healing, with the relative wound area persisting at 12.2% of its initial inflicted size Fig. 13.

It is worth mentioning that the groups treated with AgNPs-LTF gel had an accelerated healing rate compared to those treated with only LTF gel^77^. This phenomenon can be attributed to AgNPs, which enhance the antibacterial efficacy and anti-inflammatory properties of the AgNPs-LTF complex^40^. The body weight of all rats was measured before, during, and after the implementation of the treatment protocol to assess the rat’s general physiological condition^78^. The rat exhibited a significant drop in body weight following the infliction of wounds immediately preceding the initiation of the treatment protocol.

The relative body weight of the groups treated with LTF and AgNPs-LTF gel exhibited a slight recovery or remained relatively stable for 15 days. This phenomenon can be attributed to the sluggish rate of wound healing, which aligns with the findings on the size of the wound area. The body weight of the remaining groups exhibited a slight recovery to its original level before the commencement of the treatment for 15 days. However, despite the topical nature of the treatment, additional assessments were conducted to evaluate potential toxicity. These assessments included analyzing blood samples for liver enzymes (SGOT and SGPT) and renal function (Creatinine and Blood Urea Nitrogen) to ascertain any adverse effects. The results reveal that all tests fell within the usual range, suggesting that the amount of AgNPs-LTF that might be introduced into the bloodstream is non-toxic. Table 2 is a visual representation of data that presents information in a structured and organized manner.

**Table 2:** Liver enzymes and renal function for group (I) a control gel, Group( II) a gel containing only LTF; Group ( III) a gel containing AgNPs-LTF (1mM), Group( IV) a gel containing AgNPs-LTF (2mM), and Group (V) a gel containing AgNPs-LTF (4Mm), and Group( VI) Silver sulfadiazine cream from the national market.

Blood tests	Average of the groups					
	G ( I)	G( II)	G( III)	G( IV)	G(V)	G(VI)
Creatinine(mg/dl)	0.7 ± 0.1	0.7 ± 0.2	0.3 ± 0.1	0.4 ± 0.1	0.5 ± 0.1	0.6 ± 0.1
BUN(mg/dl)	20.1 ± 3.1	18.6 ± 2.3	16.2 ± 0.1	17.5 ± 0.1	18.0 ± 0.1	18.2 ± 0.1
S.GOT(IU/L)	15.7 ± 2.3	12.2 ± 0.1	8.9 ± 0.1	9.2 ± 0.1	10.5 ± 0.1	14.6 ± 0.1
S.GPT(IU/L)	18.4 ± 3.4	16.6 ± 4.1	9.73 ± 0.1	11.07 ± 0.1	13.42 ± 0.1	15.4 ± 0.1

Average normal of SGOT level is 6–30 IU/L.

Average normal of SGPT level is 6–45 IU/L.

Average normal of creatinine level is 0.2–0.8 mg/100 ml or mg/dl.

Average normal of blood urea nitrogen level is 15–21 mg/100 ml or mg/dl.

## Conclusion

This study synthesized AgNPs in the presence of LTF, forming AgNPs-lactoferrin complexes (AgNPs-LTF) across varying concentrations (1, 2, and 4 mM). LTF, a glycoprotein, played a pivotal role in precisely controlling the shape and size of the AgNPs. A stable SPR band at 427 nm in the UV-visible range provided clear evidence of the successful synthesis of AgNPs. Notably, the measured zeta potentials of + 19.6 mV for LTF and − 21.7 mV for AgNPs-LTF suggested a more substantial negative charge on the surface of AgNPs. The reduction in size from 228.2 nm for LTF to 25.81 nm for AgNPs-LTF, facilitated by LTF, holds immense promise for various applications. Remarkably, the formulation of 4 mM AgNPs-LTF exhibited remarkable antibacterial efficacy against multiple bacterial species and demonstrated a significant release of AgNPs from the gel matrix. Moreover, the AgNPs-LTF gel (4 mM) displayed notable anti-inflammatory potential, achieving an 82.7 ± 1.9% efficacy. Regarding wound healing, the wounds treated with the 4 mM AgNPs-LTF gel showed remarkable progress, with closure observed by the 9th day and complete healing by the 10th day. Noteworthy observations included the onset of hair regrowth near the wound site, indicating low cytotoxicity of the AgNPs-LTF gel (4 mM) to skin tissue. Comparative analysis showed that the LTF-treated group exhibited slightly better behavior than the control group, with the control group having an average remaining wound area of 28.34% by the 10th day. These findings collectively indicate that the AgNPs-LTF gel, especially at a concentration of 4 mM, holds considerable potential as an effective wound dressing for cutaneous wounds.

## Data Availability

The author confirms that the data supporting this study are available within the article.
